# RepE Monomer–Dimer Equilibrium Shapes Replication and Autoregulatory Control of the F Plasmid

**DOI:** 10.3390/microorganisms14030655

**Published:** 2026-03-13

**Authors:** Ján Krahulec

**Affiliations:** Department of Molecular Biology, Faculty of Natural Sciences, Comenius University Bratislava, 84215 Bratislava, Slovakia; jan.krahulec@uniba.sk

**Keywords:** plasmid replication, replication regulation, plasmid copy number control, transcriptional regulation, F plasmid

## Abstract

Although the replication mechanism of the F plasmid and its regulatory strategies have been addressed in several studies, a comprehensive understanding of these processes remains incomplete. In this work, we present new observations that contribute to refining the current model of F plasmid replication control. In this work, the results indicate that plasmid copy number control in both the F plasmid and its derivatives is consistent with two previously proposed mechanisms: the titration model and the loop formation model. In both cases, the intracellular concentration and functional state of the RepE protein appear to play a central role. Consistent with earlier reports, the data of this study support the conclusion that the RepE monomer functions as the active replication initiator. Importantly, the transcriptional analyses suggest that not only RepE dimers but also monomers contribute to autoregulatory control of *rep*E expression. These findings support a model in which the monomer–dimer equilibrium of RepE shapes both replication initiation and transcriptional autoregulation of the F plasmid.

## 1. Introduction

Replication in general occurs with the assistance of a multiprotein complex termed the replisome, which forms an open complex, synthesizes the primer, and coordinately produces the leading and lagging DNA strands with high speed and accuracy. Docking of the replisome at the initiation of replication is typically mediated by an initiator protein or multiprotein complex. The initiator recognizes and binds to specific or preferential DNA sites (origins), induces local melting of the DNA, and facilitates replisome loading through protein–protein interactions [1]

Initiators can also recognize plasmid and viral replicons, and, similar to DnaA, create an open complex at the corresponding origins, thereby allowing the replisome to bind to this structure [2,3,4]. Formation of the open complex is mediated by specific binding of the initiator to repeated sequences (iterons), followed by oligomerization and potential local deformations of DNA, such as bending, untwisting, and folding. The generation of such structures also requires DnaA, as well as the assistance of cofactors including structural proteins (IHF, Fis, HU, SSB) and structural DNA determinants, such as intrinsically bent DNA, AT-rich sequences, inherently unstable sequences like DNA unwinding elements, or DNA supercoiling [5,6].

Two main theories related to plasmid replication regulation have been proposed. The first describes control mediated by antisense RNA, as observed in ColE1 plasmids, where the antisense RNA is complementary to the primer RNA that plays a key role during replication initiation [7,8,9,10]. The second involves control mediated by short repeated sequences (iterons) localized in the origin and *Inc*C region. By binding the replication initiation protein, these iterons generate secondary structures through which replication is regulated [11,12,13,14,15,16,17].

In many cases, the replication initiation protein is present in a dimeric form that is unable to initiate replication. A crucial condition for initiation is the conversion of this protein into its monomeric form by the action of the chaperones DnaJ, DnaK, and GrpE, accompanied by energy released from ATP hydrolysis [18,19,20]. Furthermore, plasmids derived from the F plasmid are unable to replicate in cells deficient in these chaperones [20,21].

The F plasmid represents an example of replication-inactive dimers being converted into replication-active monomers through the activity of chaperones. The strict control of replication initiation in this plasmid results in the presence of only a single copy of the F plasmid per cell [22].

The replication origin used in this study, referred to as *ori*S (and in some other studies as ori2), is composed of two DnaA boxes, where the initiation protein DnaA encoded by the host genome binds. This region is characterized by a high AT content, followed by a 13-mer consensus sequence where the initial melting of the DNA strands occurs [23] and the open complex is formed. Additionally, four iterons are present, to which the initiator protein RepE binds. The gene encoding the replication protein RepE is located downstream of *ori*S. The dimeric form of RepE also binds to two inverted repeats situated between the promoter and the open reading frame of the repE gene, thereby autoregulating its own synthesis during transcription [24].

The *Inc*C locus, which also contains iterons (five in total), is located downstream of the open reading frame of the repE gene and functions as a negative regulator of F plasmid replication by controlling plasmid copy number as well as acting as an incompatibility factor [22,24,25,26,27,28]. The replication initiation protein RepE is believed to bind iterons within the *ori*S both as a dimer and as a monomer, while simultaneously binding iterons within *Inc*C, thereby creating a loop. This loop likely blocks replication initiation entirely [23,29]. Deletion of the *Inc*C locus results in an increased copy number of the F plasmid and its derivatives [26].

In this study, several plasmid constructs were generated to elucidate specific aspects of F plasmid replication regulation. The replication origin *ori*S was separated from the *rep*E gene, and these two elements were maintained in cells on separate plasmids. The primary focus was placed on the transcriptional activity of the *rep*E gene, the extent of its autoregulation, and its relationship to the copy number of the plasmid carrying *ori*S.

## 2. Material and Methods

Primers used for plasmid construction in this study are listed in Table 1. Primers used for quantitative analyses are listed in Table 2. Plasmids used in this study are listed in Table 3. *E. coli* strains used in this study are listed in Table 4.

### 2.1. Bacterial Cultivation Conditions

All strains were cultivated in LB (Luria–Bertani; 1% (*w*/*v*) enzymatic casein hydrolysate, 0.5% (*w*/*v*) yeast autolysate, 0.5% (*w*/*v*) NaCl, pH 7.2)) medium supplemented with the appropriate antibiotics (ampicillin, 50 mg/L; chloramphenicol, 5 mg/L) at 37 °C in an orbital shaker set at 150 rpm. Prior to experimental procedures, strains were streaked on LB agar plates and incubated at 37 °C from glycerol stocks stored at −70 °C to ensure efficient revitalization. A single colony-forming unit (cfu) was inoculated into 3 mL of LB medium supplemented with the corresponding antibiotic in 12-well microbiological plates. The cultures were incubated overnight under shaking conditions, after which optical density was measured and recorded. A 0.5 mL aliquot of each culture was centrifuged, and the resulting pellet was stored at −20 °C for further plasmid copy number determination or transcriptional analysis.

For cloning procedures, plasmids were isolated directly from 2 mL aliquots of overnight cultures. When plasmid copy number or transcriptional activity was assessed at the exponential growth phase, overnight cultures were used to inoculate fresh 12-well plates containing LB medium supplemented with the corresponding antibiotic at a 1:100 inoculation ratio. Cultures were incubated under the same conditions until they reached an OD600 of approximately 1. Optical density was recorded, and samples were processed analogously to stationary-phase cultures, except that cells were harvested from 1 mL volumes.

### 2.2. Molecular Cloning and Nucleic Acid Manipulations

Plasmid DNA was isolated from cultures at both growth phases using the NucleoSpin Plasmid kit (Macherey–Nagel, Düren, Germany) according to the manufacturer’s protocol. The resulting plasmid preparations were used for plasmid copy number determination by densitometry.

For plasmid copy number determination by qPCR, total genomic DNA was isolated from cultures at both growth phases using the Genomic DNA Kit Blood/Cultured Cell (Geneaid Biotech Ltd., New Taipei City, Taiwan) according to the manufacturer’s protocol with minor modifications. Cell pellets were resuspended in 300 µL of RBC buffer (kit component) and supplemented with 200 mg of 0.5 mm diameter glass beads (BioSpec Products, Bartlesville, OK, USA). Suspensions were vortexed for 1 h at 3500 rpm using a Multi Speed Vortex MSV-3500 (Biosan, Riga, Latvia). Following vortexing, suspensions were centrifuged, and 150 µL of the supernatant was processed according to the kit protocol.

For transcriptional activity analysis, total RNA was isolated from stored cell pellets using the Direct-zol RNA MiniPrep Plus kit (Zymo Research) according to the manufacturer’s protocol with minor modifications. Pellets were resuspended in 500 µL of TRI Reagent (kit component) and supplemented with 200 mg of 0.5 mm diameter glass beads (BioSpec Products, Bartlesville, OK, USA). Suspensions were vortexed for 1 h at 3500 rpm on the MSV-3500 vortex mixer (Biosan, Riga, Latvia). After centrifugation, 300 µL of the supernatant was processed according to the kit protocol.

During cloning procedures, plasmid DNA was digested with restriction endonucleases (NEB) according to the manufacturer’s instructions. Ligation reactions were performed with T4 DNA ligase (Thermo Scientific, Waltham, MA, USA) following the manufacturer’s protocol, using a vector-to-insert molar ratio of 1:5. Amplification reactions were carried out with Q5 Hot Start High-Fidelity DNA Polymerase (NEB) under the manufacturer’s recommended conditions.

Quantitative PCR reactions were performed with HOT FIREPol^®^ EvaGreen^®^ qPCR Supermix (Solis BioDyne, Tartu, Estonia) according to the manufacturer’s instructions. Reverse transcription and cDNA synthesis were performed with the FIREScript RT cDNA Synthesis Mix with Random Primers (Solis BioDyne, Tartu, Estonia) following the manufacturer’s protocol.

### 2.3. Plasmid Copy Number Determination by Densitometric Analysis

For densitometric analysis, plasmid DNA samples were separated by agarose gel electrophoresis and subsequently stained with ethidium bromide. Visualization and densitometric quantification were performed using the G:Box imaging system, and peak area analysis was carried out with Gene Tools software (both Syngene, Cambridge, UK). Supercoiled DNA Ladder (New England Biolabs, Ipswich, MA, USA) was used as the reference standard.

The following variables were included in the calculation of plasmid copy number:

- OD600 of the culture;
- Culture volume;
- Elution volume after plasmid isolation;
- Sample volume applied to agarose gel;
- Size (bp) of the analyzed plasmid;
- Peak area of the plasmid band after densitometry;
- Declared recovery rate of the plasmid isolation kit;
- Applied volume of the DNA standard;
- Declared concentration of the DNA standard;
- Declared number of base pairs of the DNA standard;
- Peak area of the standard band after densitometry.

The following constants were applied:

- Cell count at OD600 = 1: 1.6 × 109 cells/mL;
- Avogadro’s constant: 6.022 × 1023;
- Molecular mass of DNA: (number of base pairs × 617.96) + 36.04 (NEBioCalculator, NEB).

### 2.4. Quantitative PCR (qPCR) and Droplet Digital PCR (ddPCR)

qPCR assays were performed using the qTOWER^3^ Real-Time Thermal Cycler, and cycle threshold (Ct) values were determined with the qPCRsoft 4.0 software (Analytik Jena, Jena, Germany). Data analysis was carried out according to the mathematical model for relative quantification in real-time RT-PCR (Pfaffl, 2001) [30], with the modification that four housekeeping genes were used as internal standards: *cys*G (uroporphyrinogen III C-methyltransferase), *hca*T (3-phenylpropionate transporter), *rrs*A (16S ribosomal RNA), and *rpo*A (α-subunit of RNA polymerase). The geometric mean of these housekeeping genes was applied for normalization. As targets, *ori*S DNA was used to determine the plasmid copy number, and *rep*E cDNA was analyzed to assess the transcriptional activity of replication genes.

ddPCR assays were performed on the QX200 Droplet Digital PCR system (Bio-Rad Laboratories, Hercules, CA, USA). Droplet generation and reading were performed with the QX200 Droplet Reader, and data were acquired using QuantaSoft 1.7.4.0917 software. Quantification was carried out using either QuantaSoft 1.7.4.0917 or QX Manager Standard Edition 2.3.0 (Bio-Rad). For ddPCR-based copy number determination, *ori*S DNA was used as the plasmid target and *cys*G DNA as the reference gene. The absolute plasmid copy number per cell was calculated as the ratio of *ori*S to *cys*G copy numbers.

## 3. Results

### 3.1. Construction of Relevant F Plasmid Derivatives

The plasmid construction schemes are provided as Supplementary Files. All plasmids described in this study were derived from the replication region of the F plasmid. The primary structures of all constructs were validated by DNA sequencing. As a first step, a mini-F plasmid was constructed and designated pFM1. Plasmid pFM1 harbors *ori*S, the gene encoding the replication initiation protein RepE, the iteron region *Inc*C, the stabilizing locus *par*B from the R1 plasmid, and the β-lactamase gene conferring ampicillin resistance.

The replication element from the F plasmid (1430 bp) was amplified by PCR using primers FPciI L and FEcoRIXbaI P, digested with restriction enzymes *Pci*I and *Eco*RI, and subsequently cloned into the 1812 bp vector backbone of plasmid pRA5 (previously constructed in our laboratory, unpublished), which carries both the ampicillin resistance marker and the *par*B locus. The resulting construct, pFM1, is 3243 bp in length and served as the foundation for subsequent experiments.

Propagation of pFM1 was possible only in cells lacking the F plasmid, due to incompatibility. Attempts to transform cells already carrying any form of the F plasmid (e.g., *E. coli* XL1-Blue with F′) consistently failed to yield colonies.

The second step was to test whether the replication elements *ori*S and *rep*E, which normally act in *cis*, are also able to functionally initiate replication when separated and provided in *trans*. For this purpose, plasmid pFE1 was constructed, in which the region *ori*S is absent. Plasmid pFE1 was created by amplification of a 1169 bp fragment from pFM1 using primers EBsu36I L and FEcoRIXbaI P, followed by cloning into plasmid pRE31 (previously constructed in our laboratory, unpublished) via *Xba*I and *Bsu*36I restriction endonucleases. The resulting construct, designated pFE1, is 3266 bp in length and carries the pBR322 origin of replication, the chloramphenicol resistance gene, *rep*E, and the *inc*C region, but lacks *ori*S.

The replication origin *ori*S was placed separately on plasmid pFO1, which was constructed by PCR amplification of a 2097 bp fragment from pFM1 using primers oriSMfeI L and ampMfeI P, digested with *Mfe*I, and self-ligated. However, when *E. coli* DH5α cells harboring pFE1 were transformed with plasmid pFO1, no colonies were obtained after overnight selection on ampicillin LB agar plates. We concluded that the *inc*C region downstream of *rep*E in pFE1 inhibited replication of pFO1, most likely due to an excessive *inc*C dosage caused by the high copy number of pBR322-derived plasmids (≈20 copies per cell).

To circumvent this inhibitory effect, plasmid pFE10 was constructed, in which the *inc*C region was replaced by the transcriptional terminator *rrn*B T1. A 111 bp *rrn*B T1 fragment was amplified from pRE21 using primers rrnBrepE L and rrnBXbaI P. In parallel, the 154 bp 3′-end of the *rep*E gene was amplified from pFM1 using primers repErrnB P and repE PstI L. These two fragments were fused by PCR, digested with *Pst*I and *Xba*I, and cloned to replace the corresponding *Pst*I–*Xba*I fragment in pFE1. The resulting plasmid pFE10 was 3058 bp in size.

**Figure 1:**
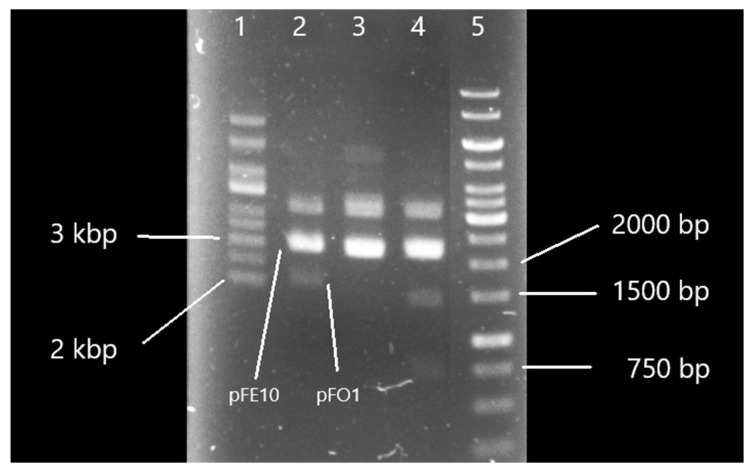
Electrophoresis of plasmids pFE10 and pFO1. Lane 1: supercoiled DNA ladder; Lane 2: pFE10, pFO1; Lane 3: pFE10, pFO1 digested with *Mfe*I; Lane 4: pFE10, pFO1 digested with *Bgl*II + *Pvu*I; Lane 5: linear DNA ladder.

Transformation of *E. coli* DH5α cells carrying pFE10 with pFO1 yielded a high number of colonies (>1000 cfu) on ampicillin LB agar. Plasmid isolation followed by restriction analysis with enzymes specific for pFO1 (*Mfe*I producing a single 2097 bp fragment; *Bgl*II + *Pvu*I generating fragments of 1389 bp and 708 bp) confirmed the coexistence of both plasmids (Figure 1). On agarose gel electrophoresis, the linear form of pFO1 (2097 bp) partially overlapped with the supercoiled form of pFE10 (3058 bp), visible as a slightly lower band in lane 3 compared to lanes 2 and 4. Sequence analysis further validated the identity of pFO1 and confirmed the restriction analysis results.

From the data of Zzaman and Bastia [29], which demonstrated that RepE exists in cells in different ratios of monomeric and dimeric forms in wild-type and mutated (R118P) variants, we constructed plasmid pFE11 to investigate the impact of this mutation on the copy number of pFO1 and on the transcriptional activity of the *rep*E gene. This publication [29], together with several earlier reports [21,31,32], postulated that the dimeric form of RepE serves mainly as its own repressor, whereas the monomeric form acts primarily as an initiator of replication.

Plasmid pFE11 was derived from plasmid pFE10. Using overlapping mutagenic primers repE118 R and repE118 F, side primers EBsu36I L and repErrnB P, and fusion PCR technology, an 875 bp fragment carrying the desired mutation was amplified. The wild-type region of pFE10 was then replaced by the mutated fragment using the restriction endonucleases *Eco*NI and *Pst*I, generating plasmid pFE11 of identical size but carrying the R118P substitution. Plasmids pFE10 and pFE11 could also be distinguished by restriction digestion with *Fsp*AI.

**Figure 2:**
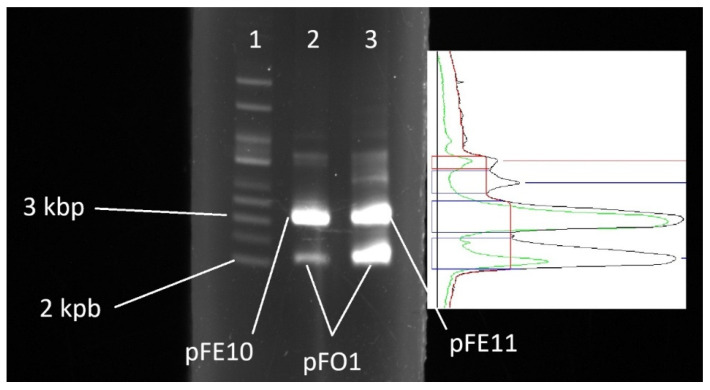
Electrophoresis (**left**) and densitometric profile (**right**) of plasmids pFE10/pFO1 and pFE11/pFO1. Lane 1: supercoiled DNA ladder; Lane 2: pFE10 and pFO1 (green line in the densitometric profile corresponds to lane 2 of electroforesis); Lane 3: pFE11 and pFO1 (black line in the densitometric profile corresponds to lane 3 of electroforesis). Red line corresponds to the baseline for integration.

*E. coli* DH5α cells harboring plasmid pFE11 were transformed with plasmid pFO1, and after selection on ampicillin LB agar plates, a substantial number of colonies (over 1000 cfu) were observed, similar to the case of cells carrying plasmid pFE10. Following plasmid isolation, a marked difference in the copy number of plasmid pFO1 was observed between cells harboring pFE10 and those harboring pFE11 (Figure 2).

### 3.2. Plasmid Copy Number Determination

Three independent methods were employed for plasmid copy number determination. For all experiments, the results from at least four measurements were taken into account, mostly ranging from six to nine replicates. The first method was densitometry of visualized agarose gels, the second was qPCR, and the third was ddPCR. Detailed descriptions of these methods are provided in the Section 2.

Plasmid copy number was determined under two different growth conditions: during the exponential phase (OD600 ≈ 1) and in the stationary phase (after overnight cultivation, OD600 ≈ 4). qPCR and ddPCR analyses were performed for plasmids F, pFM1, and pFO1, while densitometry was applied only to plasmids pFM1 and pFO1, as the size, low concentration, and quality of the F plasmid after isolation limited the applicability of this method.

**Figure 3:**
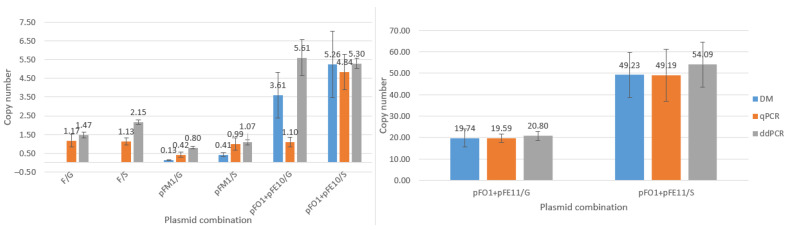
Plasmid copy numbers determined by three independent methods: densitometry (DM), qPCR, and ddPCR. Measurements were performed for plasmids *F*, pFM1, and pFO1 during exponential growth phase (OD600 ≈ 1, labeled as G) and stationary phase (overnight culture, labeled as S).

Copy number determination of plasmids F and pFM1 was included to demonstrate that the replication regulation of the mini-F plasmid pFM1 is at least comparable to that of the native F plasmid. As shown in Figure 3, which reproduces copy numbers measured by qPCR and ddPCR, both plasmids exhibited values close to one at the stationary phase, and the difference between them was not statistically significant.

Comparable results were also obtained for the *F* plasmid in cells at the exponential growth phase. In contrast, plasmid pFM1 displayed markedly lower copy numbers, oscillating around 0.42 and 0.80 as measured by qPCR and ddPCR, respectively. Consistently subunitary values were also observed for pFM1 when determined by densitometry, yielding 0.41 in stationary phase and as low as 0.13 in exponential phase.

Plasmid pFO1 was analyzed in cells harboring either pFE10 or pFE11, with copy numbers determined by all three methods: densitometry, qPCR, and ddPCR under both growth conditions. It was evident that the point mutation in the *rep*E gene (R118P substitution) had a substantial impact on the copy number of pFO1.

When co-distributed with plasmid pFE10, the average copy number of pFO1 (mean of all three methods) was 5.13 in stationary phase (≈5 copies per cell). In exponential phase, more pronounced discrepancies among the applied methods were observed, yielding 3.51, 1.10, and 5.51 copies per cell as determined by densitometry, qPCR, and ddPCR, respectively. In contrast, when pFO1 was co-distributed with pFE11, the average copy numbers reached 50.8 in stationary phase (≈51 copies per cell) and 19.7 in exponential phase (≈20 copies per cell). This corresponds to more than an eightfold increase in pFO1 copy number caused by the R118P mutation in stationary phase.

When considering methodological aspects, certain inconsistencies were noted. In all cases, copy numbers of pFO1 determined in the presence of either pFE10 or pFE11 were higher in stationary phase compared to exponential phase, regardless of the method used. However, the ratios between values obtained by the different methods varied between pFE10- and pFE11-containing cells, as well as among the three methods themselves.

### 3.3. Transcriptional Profiling

The qPCR method was also applied to determine the transcriptional activity of *rep*E in cells carrying the wild-type plasmid F, the mini-F plasmid pFM1, plasmid pFE10 without point mutation, and plasmid pFE11 carrying the R118P substitution. For all experiments, the results of at least four independent measurements were considered, with most analyses based on 6–9 replicates. Transcriptional activity was evaluated during exponential growth phase (OD600 ≈ 1) and stationary phase (overnight culture). Measurements were performed both in the absence and presence of plasmid pFO1.

**Figure 4:**
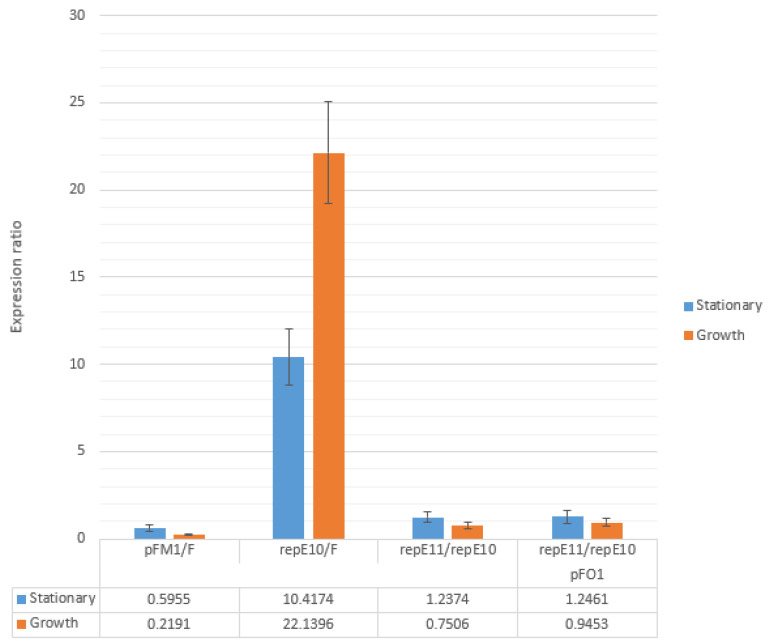
Relative *rep*E mRNA levels measured by qPCR under exponential and stationary growth phase, with and without pFO1. Relative mRNA levels were determined as the ratio of mRNA abundance in cells carrying plasmids pFM1 and repE10 relative to cells carrying plasmid F, and in cells carrying plasmid repE11 relative to cells carrying plasmid pFE10.

As shown in Figure 4, the ratio of *rep*E mRNA levels in cells carrying pFE11 compared to pFE10 was only about 24% above one, both with and without pFO1. During exponential growth, however, this ratio dropped below one under both conditions, indicating that *rep*E transcript abundance was lower in cells carrying pFE11 than in those carrying pFE10, regardless of the presence of pFO1.

Interestingly, *rep*E mRNA levels in cells harboring pFM1 were markedly lower than in cells with the F plasmid—by approximately 40% in stationary phase and nearly 80% in exponential phase. In contrast, *rep*E expression in cells with pFE10 was substantially higher compared to F plasmid, which partially corresponds to the increased copy number of pFO1 in cells carrying pFE10.

**Figure 5:**
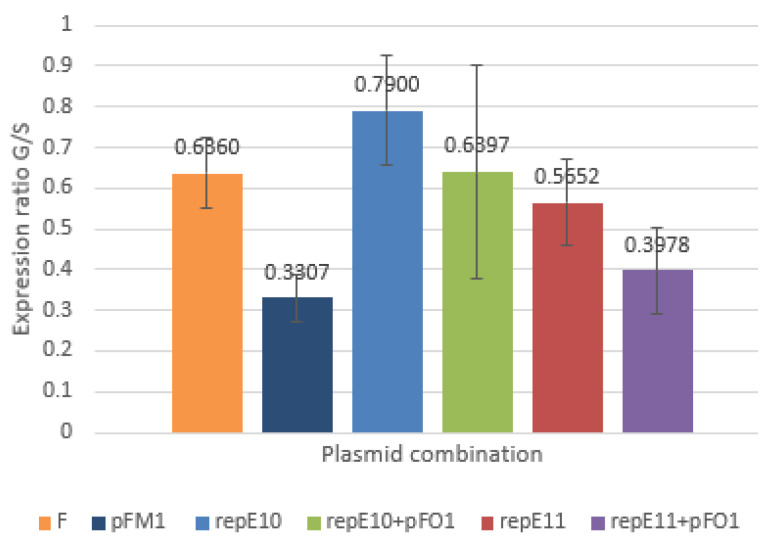
Comparison of mRNA concentrations measured during exponential growth phase (OD_600_ ≈ 1, labeled as G) and stationary growth phase (overnight culture, labeled as S) in cells carrying plasmids F, pFM1, pFE10, and pFE11, with or without the presence of plasmid pFO1.

In all cases, the concentration of mRNA for *rep*E was lower in cells at the exponential growth phase than in those at the stationary phase (Figure 5). The effect of growth phase on *rep*E transcript levels was nearly twofold stronger for the pFM1 plasmid compared to the F plasmid. The R118P mutation also had a pronounced effect: the difference in *rep*E mRNA levels between stationary and exponential phases was about 1.5 times higher for the mutant plasmid than for the wild-type plasmid, both in the presence and absence of pFO1. The presence of pFO1 itself influenced the difference in *rep*E transcript levels only moderately, with values being 1.23- and 1.42-fold higher in cells carrying pFE10 and pFE11 with pFO1, respectively, compared to the same strains without pFO1.

**Figure 6:**
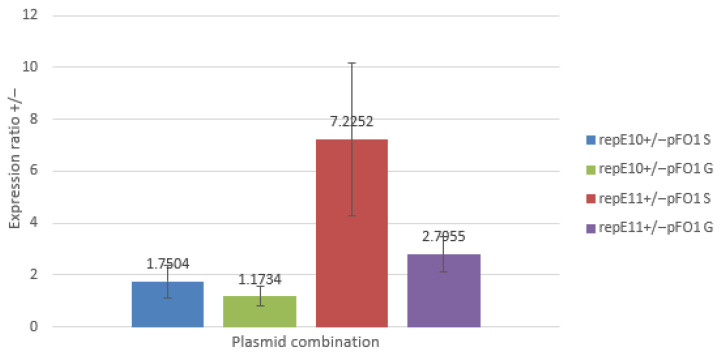
Relative mRNA concentrations were determined by qPCR. Comparison was made between cells carrying plasmids pFE10 and pFE11, either with (labeled as “+”) or without (labeled as “−”) plasmid pFO1, during exponential growth phase (OD600 ≈ 1, labeled as G) and stationary growth phase (overnight culture, labeled as S).

Concentration of mRNA for the *rep*E gene inside cells was also influenced by the presence of plasmid pFO1 (Figure 6). In all cases, the concentration of mRNA of the *rep*E gene was higher in cells carrying plasmid pFO1. The stationary phase showed higher concentrations of mRNA than the growth phase, with values about 1.5 and 2.6 times higher for cells with plasmids pFE10 and pFE11, respectively. The impact of the R118P mutation was also considerable, resulting in about 4.1- and 2.4-fold higher mRNA concentrations in stationary and growth phases, respectively.

## 4. Discussion

The initial step of plasmid replication typically involves the formation of an open complex at the replication origin, where the helicase subsequently binds to unwind the DNA double helix. In the case of the F plasmid, the replication protein RepE, together with DnaA and other proteins, plays a crucial role in replication initiation. The formation of loops at the origin between iterons is mediated by RepE molecules and represents a key element of replication regulation in the F plasmid [29,33].

Although numerous studies have been published describing the mechanism of F plasmid replication, and several models have been proposed—with many critical aspects elucidated—certain points remain unclear or even contradictory. Earlier models suggested a titration mechanism for plasmid copy number regulation [26,34], while more recent studies support the loop-formation model, where the concentration of RepE protein plays only a minor role [24,29].

The findings of this study provide new insights that may contribute to a more precise understanding of the principles underlying replication regulation of the F plasmid.

As described in the Results, several plasmids were constructed to verify previously published theories. The copy number of the mini-F plasmid pFM1 during exponential growth was consistently below one, in contrast to the native F plasmid, which exhibited values slightly above one. The mini-F plasmid pFM1 harbors only the *ori*S replication origin and the *rep*E gene from the F plasmid, but lacks additional stabilization elements of the F plasmid. Although pFM1 carries the stabilizing *par*B locus from the R1 plasmid [35], the effect of natural stabilization determinants of the F plasmid is likely stronger—particularly if replication of the F plasmid is indeed tightly coupled to cell division, as postulated by Cooper and Keasling [36]. However, this concept remains under debate, since the findings of Helmstetter et al. [37] are in conflict with the model proposed by Cooper and Keasling [36].

Similarly, as performed by Tolun and Helinsky [38], in this study the gene *rep*E encoding the replication protein RepE was separated from *ori*S and functionally propagated in cells as two different plasmids, where plasmid pFO1 with *ori*S was dependent on a plasmid carrying the *rep*E gene (pFE plasmids). Previous studies also demonstrated that replication of a plasmid carrying only *ori*S is possible in cells where the *rep*E gene is integrated into the chromosome [38].

The pFO1 plasmid copy number in the presence of pFE10 (pFE1 without *Inc*C) was determined by qPCR and densitometry to 4.84 and 5.26, respectively, at the stationary phase, which is in accordance with the study of Kawasaki et al. [39], where a similar system was used and the copy number for the plasmid carrying the *ori*S was determined to 5.3. Three mutations selected by Kawasaki et al. [39] at positions 92 and 109 had a significant impact on the copy number of the plasmid with *ori*S. The amount of RepE protein in immunoblot for mutated forms of RepE was substantially higher than for the wild-type RepE and corresponded to the copy number of mini-F plasmids carrying either mutated or wild-type *rep*E. Autoregulation repression activity was also affected by these mutations, but in a negative manner, as the mutations decreased the repression ability of RepE [39].

Notably, absolute plasmid copy numbers determined by densitometry, qPCR, and ddPCR during exponential growth phase showed quantitative differences, which likely reflect methodological limitations intrinsic to each approach. Densitometric analysis may be influenced by plasmid topology and differential migration of supercoiled versus relaxed forms, whereas qPCR depends on amplification efficiency. ddPCR, which does not require standard curves and is less sensitive to amplification efficiency bias, was therefore considered the most robust method for absolute quantification in this study.

The pFO1 plasmid copy number in the presence of pFE11 (pFE10 with mutated RepE, R118P) was determined by both methods, qPCR and densitometry, to approximately 49 copies per cell at stationary phase. Higher copy number is most likely caused by a higher concentration of monomeric RepE. Negative regulation could not be realized in this situation as the *Inc*C region is not present on either the pFO1 or pFE11 plasmids, so the plasmid copy number of pFO1 is dependent mainly on the concentration of RepE monomers. The low copy number in the case of the pFE10 plasmid and the relatively high one in the case of the pFE11 plasmid supports the theory that RepE monomers are initiators of replication and partially also the theory about the titration model postulated in some previous studies [26,34].

In contrast, although the transcriptional activity of the *rep*E11 gene (RepE with R118P) in our study was higher than the transcriptional activity of *rep*E10 (wild type RepE) during stationary phase, the difference was only about 23–24%. Moreover, during the exponential growth phase it was even lower, by about 25%. These results conflict with conclusions or models postulating that dimeric forms of RepE act as autoregulators of their own transcription, while monomeric forms act predominantly as replication initiators [32,40,41,42]. Furthermore, the transcriptional activity of both forms of the *rep*E gene (wild type and mutated) remained relatively comparable, even in the absence of the pFO1 plasmid. If only dimeric forms of RepE affected transcription of their own gene by blocking the operator, the transcriptional activity of the mutated form of *rep*E would be expected to be substantially higher. Our results therefore suggest, similarly to the model of negative regulation of plasmid copy number postulated by Zzaman and Bastia [29], that negative autoregulation of transcription is mediated by both monomeric and dimeric forms of RepE.

From the results obtained in this study, together with previously published findings, it is possible to hypothesize that both the loop and titration models play important roles in the regulation of plasmid replication. At relatively low intracellular concentrations of RepE, the negative regulatory loop cannot be formed, and the titration model becomes predominant [26,34], ensuring a smooth and gradual increase in RepE until its concentration reaches a threshold where loop formation occurs, thereby blocking both replication and transcription [29]. This may explain why plasmid pFO1 could not replicate in the presence of pFE1 (containing *inc*C), where a strong trans interaction likely occurred between pFO1 and pFE1 mediated by both monomeric and dimeric forms of RepE. Since pFE1 is present at approximately 20 copies per cell, the total concentration of RepE is higher than in cells with only a single copy of the *rep*E gene. Under such conditions, pFO1 plasmids would immediately interact in trans with pFE1 via RepE molecules, blocking their own replication without suppressing *rep*E transcription from pFE1. Consequently, RepE levels do not decrease, maintaining replication inhibition throughout the cell cycle. These findings partially support the loop creation model of replication regulation.

In contrast, in the presence of pFE10 and pFE11 (both lacking *inc*C), plasmid pFO1 was stably maintained at copy numbers of approximately 5 and 50, respectively, during stationary phase, highlighting the importance of the titration model. The striking difference in copy number was most likely due to the altered ratio of RepE monomers to dimers, rather than to differences in the total amount of RepE. This finding supports previous evidence that monomeric RepE acts as the replication initiator [21,23,32]. In the absence of *inc*C, the negative regulatory loop blocking replication and transcription is not realized, leaving replication control of pFO1 and transcriptional autoregulation of *rep*E fully dependent on the availability of RepE. Importantly, the balance of RepE concentration is maintained not only through autoregulatory transcription of *rep*E but also through titration by iterons in *ori*S on the pFO1 plasmid.

Based on the presented results and available literature, several hypotheses can be formulated, which are outlined in the following section. Logically, for F and mini-F plasmids, immediately after cell division, the concentration of RepE in daughter cells remains the same as it was in the mother cell. At this concentration, replication is blocked by the negative action of the loop described by Zzaman and Bastia [29], since such a level of RepE supports loop formation. Transcription is probably blocked as well, because it can be hypothesized that five iterons in *inc*C interact with four iterons in *ori*S together with one iteron located in the operator region oriented in the same direction. This interaction would completely suppress not only replication but also transcription. This hypothesis is further supported by the results of this study, showing that the intracellular concentration of *rep*E mRNA is lower during the growth phase than during the stationary phase.

As the cell grows after division, the intracellular concentration of RepE decreases until the loop is released, at which point transcription of *rep*E resumes and one round of replication is initiated. This hypothesis is consistent with the observation that replication of the F plasmid does not begin immediately after cell division, when cells are short (<1 μm), but rather when the cells reach a length between 1.4 and 1.8 μm. Replication is then blocked again until the next division [43]. Immediately after loop relaxation, replication occurs simultaneously with *rep*E transcription. During this single replication cycle, the concentration of RepE increases again to a level sufficient to re-establish loops on both plasmids, thereby blocking both replication and transcription until the following cell cycle. This hypothesis is also supported by the ddPCR results obtained in this study, where the copy number of the F plasmid was determined to be 2 copies per cell.

If the copy number of plasmid pFO1 is substantially lower during the growth phase, it means that transcriptional repression is quite strong and that RepE protein concentration increases more slowly than cell growth. This interpretation is supported by transcriptional profiling data obtained in this study and is valid for both situations, either in the presence of pFE10 or pFE11. Transcription frequency is probably indirectly dependent on the concentration of both forms of RepE protein (monomer and dimer) together, because, as mentioned earlier, the transcriptional activity of either *rep*E10 or *rep*E11 with or without pFO1 is not substantially different, whereas the copy number of pFO1 varies strongly.

Thus, autoregulation also occurs in the absence of *inc*C, mediated solely by the intracellular concentration of RepE protein, where the monomer acts as a replication initiator and both forms function as transcriptional repressors of the *rep*E gene. During intensive cell division and growth, the concentration of RepE (both forms) inside cells decreases, which correlates with the lower copy number of pFO1 plasmid. At the same time, repression of *rep*E weakens and transcription becomes more intensive. In this phase, a dynamic balance is established between transcriptional activation and repression. Although the transcription rate slightly lags behind cell growth and division, autoregulation maintains the balance and prevents plasmid segregation. These results provide clear evidence of autoregulation, in which RepE concentration is inversely proportional to *rep*E transcription.

Kawasaki et al. [39] demonstrated that at maximum level of repression, when the *rep*E gene was placed under the control of a tryptophan promoter and the *lac*Z gene under the control of the *rep*E promoter, the expression level in the absence of tryptophan reached only 8% of the unrepressed level. With 5 mg/L of tryptophan, expression increased to 21%, but at higher concentrations only slight additional increases were observed. These findings indicate that the strength of autorepression is relatively high, since even under conditions of basal *rep*E expression from the tryptophan promoter, the expression driven by the *rep*E promoter remains low, reaching only a little over 20% of the level without repression.

Moreover, the results of Kawasaki et al. [39] show that the inverse relationship between RepE concentration and transcriptional activity is not linear, supporting the hypothesis that during intensive cell division a balance is established between autorepression and transcription. This concept of strong autorepression of the *rep*E gene is also consistent with the results of this study, where the intracellular concentration of *rep*E mRNA (both *rep*E10 and *rep*E11), measured by qPCR, was lower during exponential growth than in stationary phase. These observations confirm the notion, postulated in the previous paragraph, that transcription slightly lags behind cell growth and division.

## Figures and Tables

**Table 1:** Primers used for plasmid construction.

FPciI L	5′-CCCCACATGTCACTTATATATTCTGCTTACACACGATGCCTG-3′
FEcoRIXbaI P	5′-CCCCGAATTCTCTAGATCCAGGTAGAGGTACACACGCGG-3′
EBsu36I L	5′-CCCCCCTGAGGGCTGTTTTTTCACAAAGTTATCCCTGCTTATTGAC-3′
oriSMfeI L	5′-CCCCCAATTGCTTTGTGAAAAAACAGCTTCTTCTGAGGGC-3′
ampMfeI P	5′-GGGGCAATTGGAAATGTGCGCGGAACCCCTATTTG-3′
rrnBrepE L	5′-GACGACAGGATAGCAAATAAAACGAAAGGCTCAGTCGAAAGACTG-3′
rrnBXbaI P	5′-CCCCTCTAGAATTTGTCCTACTCAGGAGAGCGTTCACC-3′
repErrnB P	5′-GCCTTTCGTTTTATTTGCTATCCTGTCGTCATGGAAGTGATA TCGC-3′
repE PstI L	5′-CTTCCTGCAGGTCTGTGTTAATGAGATC-3′
repE118 R	5′-TGGACTGTGCGCAGGTTTGATAAACCAAGG-3′
repE118 F	5′-CCTTGGTTTATCAAACCTGCGCACAGTCCA-3′

**Table 2:** Primers used for quantitative analysis.

RTF cysG	5′-TTAATCAACGAACCGCTCGACCATC-3′
RTR cysG	5′-TCGTAGACCACCACATCTGCCT-3′
RTF rrsA	5′-CTGGGAACTGCATCTGATACTGG-3′
RTR rrsA	5′-CTTCGTCCAGGGGGCCG-3′
RTF rpoA	5′-GGTTATGTGCCGGCTTCTACC-3′
RTR rpoA	5′-ACACGCGCTGCTTCAACATTGTAG-3′
RTF hcaT	5′-TGGCTCGGCGCTGACGG-3′
RTR hcaT	5′-CTTGTGGCTGAATCGTCGGAC-3′
RTF repE	5′-CCGCGAATCGTCCAGTCAAAC-3′
RTR repE	5′-TCATGTTCCTGTSGGGTGCCATC-3′
RTF oriS	5′-ACAGACAGGACTGTCATTTGAGGGTGATT-3′
RTR oriS	5′-TTTTTAGATCTTCTTTTTTAGAGCGCCTTGTAGG-3′

**Table 3:** Plasmids used in this study.

F′	proAB lacI^q^Z∆M15 Tn10 (Tet^r^)	Part of XL1-Blue Cells
pRA5	4209bp, Amp^R^, *par*B, *cop*B, *rep*A1, R1 origin, *rep*A4	created in our laboratory, not published
pRE31	3811 bp, Cm^R^, pBR322 ori, *cop*B, *rep*A1	created in our laboratory, not published
pFM1	3243 bp, Amp^R^, *par*B, *rep*E, *Inc*C, *ori*S	created in this work
pFE1	3266 bp, Cm^R^, pBE322 ori, *rep*E, *Inc*C	created in this work
pFE10	3058 bp, Cm^R^, pBR322 ori, *rep*E, *rrn*B	created in this work
pFE11	3058 bp, Cm^R^, pBR322 ori, *rep*E(R118P), *rrn*B	created in this work
pFO1	2097bp, Amp^R^, *par*B, *ori*S	created in this work

**Table 4:** *E. coli* strains in this study.

XL1-Blue	*rec*A1 *end*A1 *gyr*A96 thi-1 *hsd*R17 *sup*E44 *rel*A1 *lac* [F′ *pro*AB *lac*IqZ∆M15 Tn*10* (Tet^r^)]
DH5α	F^–^ *end*A1 *gln*V44 thi-1 *rec*A1 *rel*A1 *gyr*A96 *deo*R *nup*G *pur*B20 φ80d*lac*ZΔM15 Δ(*lac*ZYA-*arg*F)U169, *hsd*R17(r_K_^–^m_K_^+^), λ^–^

## Data Availability

The original contributions presented in this study are included in the article/Supplementary Materials. Further inquiries can be directed to the corresponding author.

## Supplementary Materials

The following supporting information can be downloaded at: https://www.mdpi.com/article/10.3390/microorganisms14030655/s1.

## References

[1] Le Chatelier E., Jannie L., Ehrlich D., Canceill D. (2001). The RepE Initiator Is a Double-stranded and Single-stranded DNA-binding Protein That Forms an Atypical Open Complex at the Onset of Replication of Plasmid pAMb1 from Gram-positive Bacteria. J. Biol. Chem..

[2] del Solar G., Giraldo R., Ruiz-Echevarria M.J., Espinosa M., Diaz-Orejas R. (1998). Replication and control of circular bacterial plasmids. Microbiol. Mol. Biol. Rev..

[3] Khan S.A., Chattoraj D.K. (1998). Initiation of DNA replication in phages and plasmids-a workshop summary. Plasmid.

[4] DePamphilis M.L. (1996). DNA Replication in Eukaryotic Cells.

[5] Zannis-Hadjopoulos M., Price G.B. (1999). Eukaryotic DNA replication. J. Cell. Biochem..

[6] Boulikas T. (1996). Common structural features of replication origins in all life forms. J. Cell. Biochem..

[7] Itoh T., Tomizawa J. (1980). Formation of an RNA primer for initiation of replication of ColE1 DNA by ribonuclease H. Proc. Natl. Acad. Sci. USA.

[8] Polisky B. (1988). ColE1 replication control circuitry: Sense from antisense. Cell.

[9] Tomizawa J. (1990). Control of ColE1 plasmid replication. Interaction of Rom protein with an unstable complex formed by RNA I and RNA II. J. Mol. Biol..

[10] Tomizawa J. (1990). Control of ColE1 plasmid replication. Intermediates in the binding of RNA I and RNA II. J. Mol. Biol..

[11] Filutowicz M., McEachern M., Greener A., Mukhopadhyay P., Uhlenhopp E., Durland R., Helinski D., Hollaender A. (1985). Role of the π initiation protein and direct nucleotide sequence repeats in the regulation of plasmid R6K replication. Plasmids in Bacteria.

[12] Germino J., Bastia D. (1982). Primary structure of the replication initiation protein of plasmid R6K. Proc. Natl. Acad. Sci. USA.

[13] Germino J., Bastia D. (1983). The replication initiator protein of plasmid R6K tagged with b-galactosidase shows sequence specific DNA binding. Cell.

[14] Germino J., Bastia D. (1983). Interaction of plasmid R6K encoded replication initiator protein with its binding site on DNA. Cell.

[15] Stalker D.M., Kolter R., Helinski D.R. (1982). Plasmid R6K DNA replication. I. Complete nucleotide sequence of an autonomously replicating segment. J. Mol. Biol..

[16] McEachern M., Bott M.A., Tooker P.A., Helinski D.R. (1989). Negative control of plasmid R6K replication: Possible role of intermolecular coupling of replication origins. Proc. Natl. Acad. Sci. USA.

[17] Pal S.K., Chattoraj D.K. (1988). P1 plasmid replication: Initiator sequestration is inadequate to explain control by initiator-binding sites. J. Bacteriol..

[18] Pak M., Wickner S. (1997). Mechanism of protein remodeling by ClpA chaperone. Proc. Natl. Acad. Sci. USA.

[19] Wickner S., Skowyra D., Hoskins J., McKenney K. (1992). DnaJ, DnaK, and GrpE heat shock proteins are required in oriP1 DNA replication solely at the RepA monomerization step. Proc. Natl. Acad. Sci. USA.

[20] Kawasaki Y., Wada C., Yura T. (1990). Roles of *Escherichia coli* heat shock proteins DnaK, DnaJ and GrpE in mini-F plasmid replication. Mol. Gen. Genet. MGG.

[21] Ishiai M., Wada C., Kawasaki Y., Yura T. (1994). Replication initiator protein RepE of mini-F plasmids: Functional differentiations between monomers (initiator) and dimers (autogenous repressor). Proc. Natl. Acad. Sci. USA.

[22] Manis J.J., Kline B.C. (1978). F plasmid incompatibility and copy number genes: Their map locations and interactions. Plasmid.

[23] Kawasaki Y., Matsunaga F., Kano Y., Yura T., Wada C. (1996). The localized melting of mini-F origin by the combined action of the mini-F initiator protein (RepE) and HU and DnaA of *Escherichia coli*. Mol. Gen. Genet. MGG.

[24] Uga H., Matsunaga F., Wada C. (1999). Regulation of DNA replication by iterons: An interaction between the ori2 and incC regions mediated by RepE-bound iterons inhibits DNA replication of mini-F plasmid in *Escherichia coli*. EMBO J..

[25] Murotsu T., Matsubara K., Sugisaki H., Takanami M. (1981). Nine unique repeating sequences in a region essential for replication and incompatibility of the mini-F plasmid. Gene.

[26] Tsutsui H., Fujiyama A., Murotsu T., Matsubara K. (1983). Role of nine repeating sequences of the mini-F genome for expression of F-specific incompatibility phenotype and copy number control. J. Bacteriol..

[27] Kline B.C., Trawick J. (1983). Identification and characterization of a second copy number control gene in mini-F plasmids. Mol. Gen. Genet. MGG.

[28] Wada C., Yura T. (1984). Control of F plasmid replication by a host gene: Evidence for interaction of the em/A gene product of *Escherichia colt* with the mini-F ineC region. J. Bacteriol..

[29] Zzaman S., Bastia D. (2005). Oligomeric Initiator Protein-Mediated DNA Looping Negatively Regulates Plasmid Replication In Vitro by Preventing Origin Melting. Mol. Cell.

[30] Pfaffl M.W. (2001). A new mathematical model for relative quantification in real-time RT-PCR. Nucleic Acids Res..

[31] Ishiai M., Wada C., Kawasaki Y., Yura T. (1992). Mini-F plasmid mutants able to replicate in *Escherichia coli* deficient in the DnaJ heat shock protein. J. Bacteriol..

[32] Matsunaga F., Kawasaki Y., Ishiai M., Nishikawa K., Yura T., Wada C. (1995). DNA-Binding Domain of the RepE Initiator Protein of Mini-F Plasmid: Involvement of the Carboxyl-Terminal Region. J. Bacteriol..

[33] Zzaman S., Abhyankar M.M., Bastia D. (2004). Reconstitution of F Factor DNA Replication in Vitro with Purified Proteins. J. Biol. Chem..

[34] Trawick J.D., Kline B.C. (1985). A two-stage molecular model for control of mini-F replication. Plasmid.

[35] Gerdes K. (1988). The parB (hok/sok) Locus of Plasmid R1: A General Purpose Plasmid Stabilization System. Nat. Biotechnol..

[36] Cooper S., Keasling J.D. (1998). Cycle-specific replication of chromosomal and F-plasmid origins. FEMS Microbiol. Lett..

[37] Helmstetter C.E., Thornton M., Zhou P., Bogan J.A., Leonard A.C., Grimwade J.E. (1997). Replication and Segregation of a miniF Plasmid during the Division Cycle of *Escherichia coli*. J. Bacteriol..

[38] Tolun A., Helinski D.R. (1982). Separation of the Minimal Replication Region of the F Plasmid into a Replication Origin Segment and a Trans-Acting Segment. Mol. Gen. Genet. MGG.

[39] Kawasaki Y., Wada C., Yura T. (1991). Mini-F plasmid mutants able to replicate in the absence of s32: Mutations in the *rep*E coding region producing hyperactive initiator protein. J. Bacteriol..

[40] Kline B.C. (1985). A review of mini-F plasmid maintenance. Plasmid.

[41] Muraiso K., Tokino T., Murotsu T., Matsubara K. (1987). Replication of mini-F plasmid in vitro promoted by purified E protein. Mol. Gen. Genet. MGG.

[42] Wada C., Imai M., Yura T. (1987). Host control of plasmid replication: Requirement for the σ factor σ32 in transcription of mini-F replication initiator gene. Proc. Natl. Acad. Sci. USA.

[43] Gordon G.S., Sitnikov D., Webb C.D., Teleman A., Straight A., Losick R., Murray A.W., Wright A. (1997). Chromosome and low copy plasmid segregation in *E. coli*: Visual evidence for distinct mechanisms. Cell.

